# The QTL within the *H2* Complex Involved in the Control of Tuberculosis Infection in Mice Is the Classical Class II *H2-Ab1* Gene

**DOI:** 10.1371/journal.pgen.1005672

**Published:** 2015-11-30

**Authors:** Nadezhda Logunova, Maria Korotetskaya, Vladimir Polshakov, Alexander Apt

**Affiliations:** 1 Laboratory for Immunogenetics, Central Institute for Tuberculosis, Moscow, Russia; 2 Center for Magnetic Tomography & Spectroscopy, School of Fundamental Medicine, M. V. Lomonosov Moscow State University, Moscow, Russia; 3 Department of Immunology, School of Biology, M. V. Lomonosov Moscow State University, Moscow, Russia; McGill University, CANADA

## Abstract

The level of susceptibility to tuberculosis (TB) infection depends upon allelic variations in numerous interacting genes. In our mouse model system, the whole-genome quantitative trait loci (QTLs) scan revealed three QTLs involved in TB control on chromosomes 3, 9, and in the vicinity of the *H2* complex on chromosome 17. For the present study, we have established a panel of new congenic, *MHC*-recombinant mouse strains bearing differential small segments of chromosome 17 transferred from the TB-susceptible I/St (*H2* ^j^) strain onto the genetic background of TB-resistant C57BL/6 (B6) mice (*H2* ^b^). This allowed narrowing the QTL interval to 17Ch: 33, 77–34, 34 Mb, containing 36 protein-encoding genes. Cloning and sequencing of the *H2* ^j^ allelic variants of these genes demonstrated profound polymorphic variations compare to the *H2* ^b^ haplotype. In two recombinant strains, B6.I-249.1.15.100 and B6.I-249.1.15.139, recombination breakpoints occurred in different sites of the *H2-Aβ 1* gene (beta-chain of the Class II heterodimer H2-A), providing polymorphic variations in the domain β1 of the *Aβ-*chain. These variations were sufficient to produce different TB-relevant phenotypes: the more susceptible B6.I-249.1.15.100 strain demonstrated shorter survival time, more rapid body weight loss, higher mycobacterial loads in the lungs and more severe lung histopathology compared to the more resistant B6.I-249.1.15.139 strain. CD4^+^ T cells recognized mycobacterial antigens exclusively in the context of the H2-A Class II molecule, and the level of IFN-γ-producing CD4^+^ T cells in the lungs was significantly higher in the resistant strain. Thus, we directly demonstrated for the first time that the classical *H2- Ab1* Class II gene is involved in TB control. Molecular modeling of the H2-A^j^ product predicts that amino acid (AA) substitutions in the Aβ-chain modify the motif of the peptide–MHC binding groove. Moreover, unique AA substitutions in both α- and β-chains of the H2-A^j^ molecule might affect its interactions with the T-cell receptor (TCR).

## Author Summary

Many genes of the host regulate interactions with *Mycobacterium tuberculosis* and determine the level of susceptibility to, and severity of, tuberculosis (TB). Identification of these genes and their alleles is continuing and contributes new knowledge about the host-pathogen interactions. So far, forward genetic approaches (from phenotype to gene) have identified several chromosomal segments involved in genetic control of TB in mice (quantitative trait loci—QTL), but only one particular gene, *Ipr1*, has been identified. Here, we report the identification of a second TB-controlling gene. On the basis of a pair of mouse inbred strains with polar susceptibility to TB infection (susceptible I/St and more resistant C57BL/6) we established a panel of recombinant strains carrying small segments of Chromosome 17 from I/St on the genetic background of C57BL/6. A combination of genetic mapping, gene sequencing, TB phenotypes assessment and immunological approaches demonstrates that the *H2-Ab1* gene encoding the beta-chain of the Class II heterodimer H2-A determines susceptibility to TB infection. The importance of allelic polymorphisms in Class II genes encoding antigen-presenting molecules in susceptibility to infection has been suspected. This is the first prove of this role obtained by the methods of classical forward genetics.

## Introduction

Tuberculosis (TB) remains a significant public health problem: one-third of the human population is infected with *Mycobacterium tuberculosis* (MTB) and 10% of those are at a risk of developing overt TB during their lifetime [1, 2]. Although there is growing body of evidence that the outcome of infection is modulated both by bacterial and host genetics [3, 4], genetic factors regulating susceptibility to infection, transition from latency to reactivation and severity of the disease remain largely unknown. The important role of host genetic factors in TB disease control in humans has been clearly demonstrated in numerous studies, including adoption [5], twin [6–8], genome-wide association (GWAS) [9–12], and case-control population [13–16] studies. Apart from rare cases of Mendelian susceptibility to mycobacterial diseases (MSMD) due to nonsense and missense mutations in key genes involved in protective immunity against intracellular pathogens (reviewed in [17]), the complex patterns of TB susceptibility and disease manifestations clearly correspond to a polygenic type of genetic control with numerous epistatic interactions (reviewed in [18]). Naturally, identification of TB control-relevant genes and alleles in humans remains a very difficult task which is complicated by the environmental and strain diversity, as well as by the lack of consensus in the definition of, and distinction between, clinical phenotypes.

TB infection can be readily induced in mice, and some refined mouse TB models reproduce human-like pulmonary infection with appreciable accuracy (see [19, 20] for the review). In a few independent studies employing different inbred mouse strains, the whole genome scan approach has been applied for genetic mapping of quantitative trait loci (QTL) involved in TB susceptibility and disease control [21–25]. Since different inbred strains were selected as susceptible and resistant parental prototypes, and different phenotypes (survival time post-infection, multiplication of mycobacteria in organs, dynamics of cachexia) were analyzed, it is not surprising that the genomic locations of most of the QTL reported in different studies did not coincide. In our TB models, we use I/St TB-susceptible and A/Sn or C57BL/6 TB-resistant mice as prototypes. TB-infected I/St mice differ profoundly from their more resistant counterparts by early onset of mortality, rapid body weight loss, increased mycobacterial multiplication in lungs and spleens, and exacerbated lung histopathology [26]. Whole genome scans performed in F2 and N2 generations identified three QTL on chromosomes 3, 9 and 17 whose allelic variation affected TB susceptibility [22, 23]. The QTL on chromosome 17, peaking at the D17Mit175 marker, overlaps with the location of the mouse major histocompatibility complex *Н2*. Remarkably, this locus remains the only known case of co-localization of TB-controlling QTL reported in previous studies: Kramnik and colleagues mapped a QTL within the *H2* region using a different combination of parental strains [21].

Associations of TB susceptibility/severity with the MHC polymorphic haplotypes have been previously reported both in humans [27–30] and mice [31–35]. In mice, allelic variations within the *H2* complex were shown to affect survival time following challenge, the level of T-lymphocyte-mediated delayed type hypersensitivity (DTH) response, T-cell proliferation after stimulation with mycobacterial antigens and the efficacy of BCG vaccination against tuberculosis, production of IFN-γ by mycobacteria-specific T-cells, and production of mycobacteria-specific antibodies [31, 36–39]. However, these early studies provided no information about any particular gene within the *H2* complex affecting TB immunity. Progression from a defined QTL region to a particular gene remains a major challenge: about 3,000 QTLs have been mapped in mice and rats but less than 1% of the genes have been identified at the molecular level [40]. This is especially true for the ~3.5 Mb MHC region, which provides the highest density of coding genes in the genome. Furthermore, many of these genes, which display a very high level of allelic variation and extensive linkage disequilibrium, have fundamental roles in immunity. Not surprisingly, numerous associations with several diseases for this part of the genome have been reported [41–44].

To begin identification of the gene, we have started to narrow the interval for the chromosome 17 QTL using a classical homologous recombination approach and have developed a panel of recombinant congenic mouse strains bearing different intra-*H2* segments from TB-susceptible I/St mice on the resistant B6 genetic background. Given the previous demonstration that an allelic variant of chromosome 17 QTL inherited from B6 mice determined resistance to infection [21], we decided to use these common and genetically well-characterized mice as the TB-resistant prototype strain. At the initial stage of this study, we succeeded in narrowing the region on chromosome 17 which determines the level of TB-susceptibility from 8–65Mb to 33,77–34,34 Mb [45]. Since gene sequencing data for the I/St inbred strain are unavailable from the databases, in the present study we cloned and sequenced coding parts of all genes annotated for this region using I/St cDNA. As expected, the region displayed a very high level of genetic polymorphism and only a few out of 36 genes demonstrated identical sequences for B6 and I/St. In addition, the region under study contains many genes of importance for immunity and cell biology, thus being realistic candidates for the infection control. Thus, we searched for recombination events inside the TB-controlling region and established new mouse strains narrowing the region to the 34,24–34,33Mb interval. This interval contains only five coding genes, all belonging to classical and non-classical Class II: *H2-Ob*, *H2-Aa*, *H2-Ab1*, *H2-Eb1* and *H2-Eb2*. Two recombinant strains with substantial differences in response to TB infection displayed recombination events in different parts of a single *H2-Ab* gene, which was critical for gene identification.

## Results

### Fine genetic mapping: round 1

**Fig 1:**
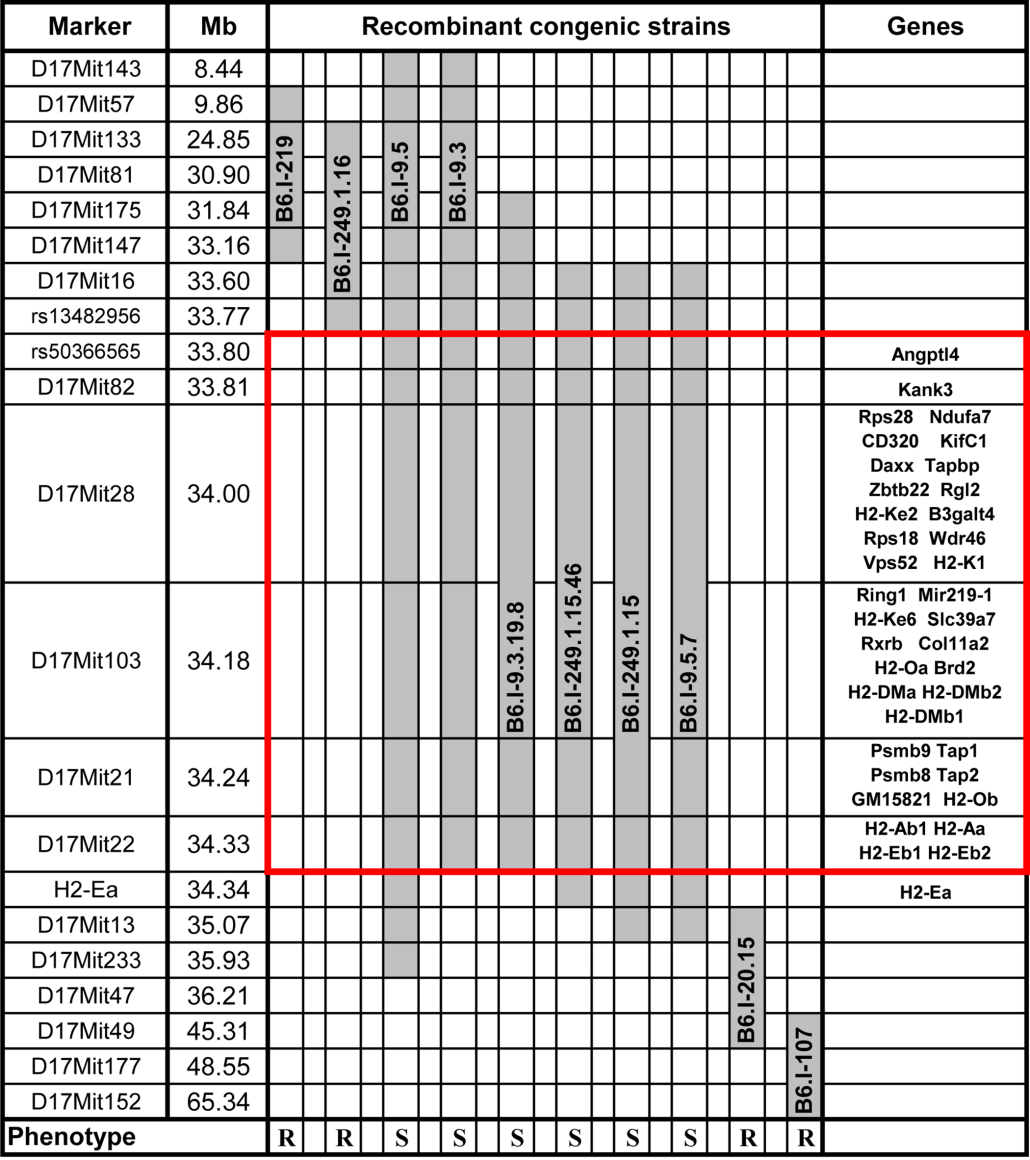
Chart of the extended *H2* genomic region in novel recombinant congenic strains. Chromosome segments transferred from I/St (*Н2* ^j^) mice onto the B6 (*Н2* ^b^) genetic background are shown in grey. Markers (all—from MGI-Mouse Genome Database (http://www.jax.org), are shown in the left column, followed by their genomic positions in mega base pairs (Mb). Gene symbols and locations (according to the Ensembl Genome assembly GRCm38.p2 (http://www.ensembl.org) are shown in the right column. All strains bearing the *Н2* ^j^-originated genetic material highlighted by the red border displayed a TB-susceptible phenotype (S, bottom), other strains were TB-resistant (R, bottom).

**Fig 2:**
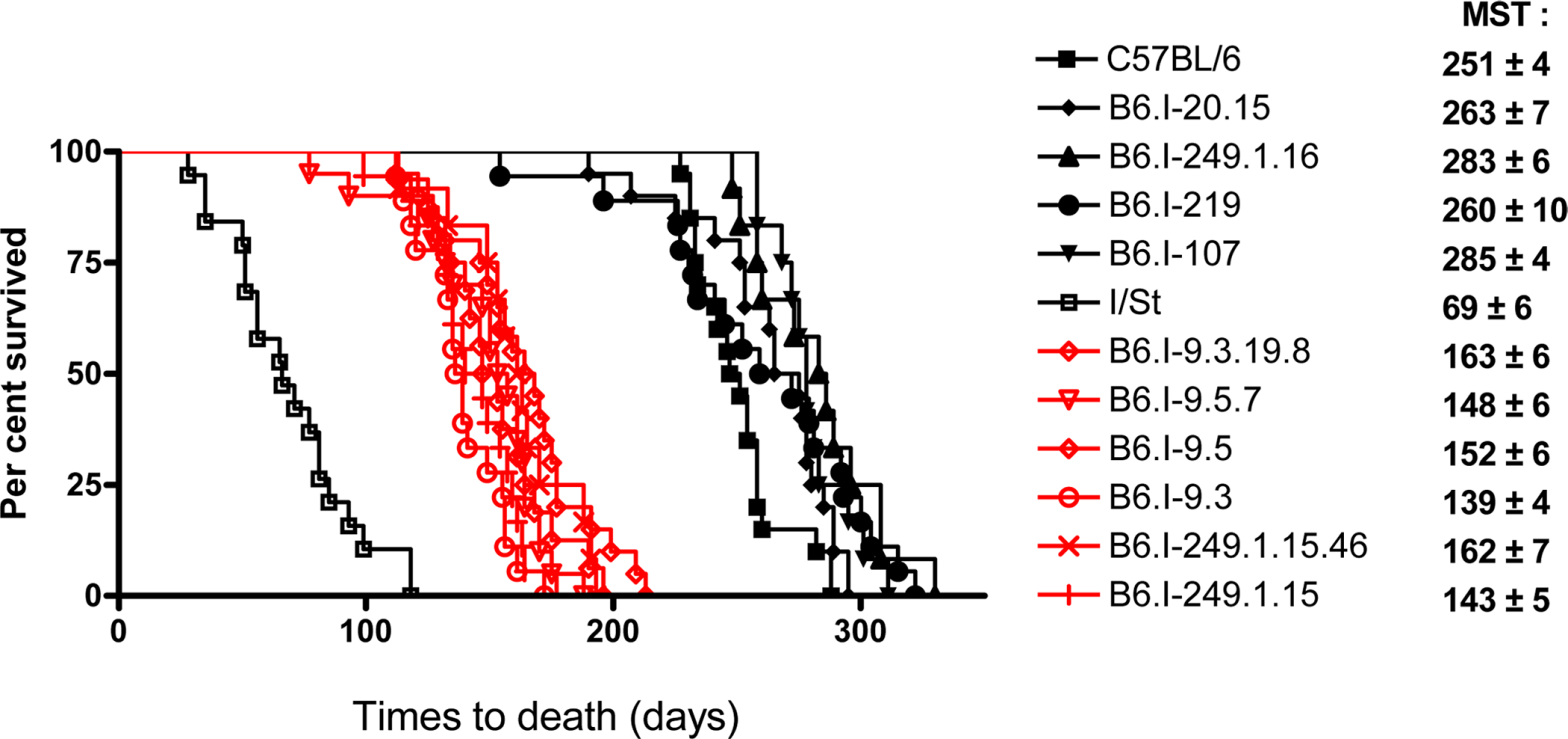
Survival curves of mice infected with *M*. *tuberculosis*. Survival of parental B6 and I/St and recombinant congenic mice (males) following aerosol challenge with ~500 CFU of *M*. *tuberculosis* H37Rv. Recombinant strains B6.I-9.3.19.8, B6.I-9.5.7, B6.I-9.5, B6.I-9.3, B6.I-249.1.15.46 and B6.I-249.1.15 all displayed similar (P > 0.1) intermediate mean survival time (MST) compared to hyper-susceptible I/St and relatively resistant B6 mice (*P* < 0.0001, log-rank test), which reflects the input of the intra-*H2* QTL in susceptibility. All recombinant strains were tested in 3–10 independent experiments (total N = 20–70 animals). Summary of 3–5 experiments is displayed (Kaplan-Meier survival analysis).

We transferred genomic regions covering the vicinity of the *H2* complex from TB-susceptible parental I/St (*H2* ^j^) mice onto the B6 (*H2* ^b^) genetic background in successive backcross generations. Starting with the BC1 (N2) generation, we applied simultaneous selection for the presence of two traits: TB-susceptible phenotype and Chromosome 17 markers of the I/St origin. At the generation N10-11, more than forty B6.I-*H2* recombinant congenic strains on the B6 background carrying different, partly-overlapping genomic regions of the extended *H2* ^j^- haplotype (17 Chr: 8,44–65,34 Mb) were generated. Fig 1 displays the most informative B6.I strains whose pheno- and genotyping allowed us to narrow the region of interest to the interval 33,77–34,34Mb (a total of 0,57 Mb). Mice of all strains which inherited this region from I/St ancestors were significantly more susceptible to infection than those bearing B6 alleles as indicated by survival curves (Fig 2) and the dynamics of cachexia (S1 Fig). Fine mapping within this region was achieved by superposition: the resistant strain B6.I-249.1.16 carries *H2* ^j^ alleles more proximal than the SNPs rs13482956 **(**17:33, 773331) whereas the strain B6.I-9.3.19.8 is susceptible although all distal genes starting with and including *H2-Ea* are of B6 origin (Fig 1). Being more susceptible than B6, all recombinant strains carrying the region 33, 77–34, 34Mb inherited from I/St were more resistant than their I/St ancestors, indicating the influence of B6 background genes on survival. This is further supported by the fact that the level of resistance was identical in parental B6 mice and in recombinant mice which inherited the identified *H2* segment from B6 (Fig 2). According to http://www.ensembl.org, the identified region contains 36 protein-coding genes, most of which may have important immunological and regulatory functions.

### Genes in the identified region are highly polymorphic

No information was available about the genome sequence of I/St mice, so it was impossible to start searching for candidate genes by direct sequence comparison. Therefore, we cloned and sequenced the protein-coding regions of all 36 I/St-originated genes in the region (GenBank, accession numbers KJ650201-KJ650234). Table 1 displays all amino acid (AA) substitutions between *H2* ^b^ and *H2* ^j^ haplotypes, as judged from the cDNA sequencing data. As expected, the region appeared to be highly polymorphic: only seven genes (*Zbtb22*, *H2-Ke2*, *B3galt4*, *Slc39a7*, *Brd2*, *H2-DMb2*, *H2-DMb1*) displayed no allelic polymorphism for the two haplotypes.

**Table 1:** Allelic polymorphisms in the coding parts of the protein-encoding genes*.

Gene	AA substitutions
*Angptl4*	N272D
*Kank3*	A324V, S580P
*Ndufa7*	A82T
*CD320*	I132M
*Kifc1*	I101V, V111A
*Daxx*	N179S, D449Del, **P504S**
*Tapbp*	**T350M**
*Rgl2*	H147Y, M402T, **P754T**
*Wdr46*	T40A, Q531K, M597K
*Vps52*	A458G
*H2-K1*	V2A, **C4G, T5K, A12V, V30Y, A32T, Y43F,** M44I, **E45S,** D51N, **D60A, Y66D,** A70V, A88S, **N91Q, E92K, Q93E, L102A,** G104R, **I116L, I119M,** S120Y, **E123D,** G128W, **Q135L, C144R, L162Q,** K165R, E173A, **R176L, L177Y, R178K, T184E, W188S, K194E, N195L,** E217K, I246T, **Y277N, Q285E,** M305T, **A306V,** T307I, V308I
*Ring1*	M160I
*H2-Ke6*	E86Q
*Rxrb*	**A51Del, A52Del, Del63E, Del64P**
*Col11a2*	**A455V,** M533V, N1239D, I1501M
*H2-Oa*	A14V, I216T
*H2-DMa*	F35L
*Psmb9*	H60R, C126R, D177N
*Tap1*	A213T, **V271M,** G277D, A414T, C544R
*Psmb8*	G272R
*Tap2*	E57G, V109A, Del136L, H353R, N424S, **D585A**
*H2-Ob*	S154N, V174I, L193H, V231A, **E265K, S266L**
*H2-Ab1*	D29N, M39K, **G40S, E41A,** Y53L, **T55S,** Y57N, **Y64F, V65M,** Y67F, **H74F, A85V,** S90K, P92Del, E93Del, I94Y, **R97Q, T98K,** E101A, L102V, W224R, H249Y
*H2-Aa*	T34S, **F51H,** L58W, A79T, **Q88K, V92T, V93G,** V99I
*H2-Eb1*	V2M, W33R, **C38S,** K39T, L53F, E55D, L61R, N64Y, **L65V, F74Y,** N87Y, **F94I, Q97D, K98A, E101S, V105Y, E214K**
*H2-Eb2*	L22V, R179G, M191T, P206L

*The *H2* ^j^ protein-encoding genes annotated for the region 34, 773331–34, 341959 were cloned and sequenced as described in Materials and Methods. Only genes with missense and non-synonymous mutations are included. Positions of (B6 → I/St) AA substitutions in the single letter code and deletions (Del) are displayed. Amino acid substitutions not reported previously (according to the Ensembl genetic variation data) are given in bold.

The *H2* segment under study contains numerous genes generally involved in immune response control, and for many of these genes evidence is available indicating their possible involvement in the control of TB infection. S1 Table briefly summarizes the data illustrating this point. In addition, alternative splicing isoforms for many I/St alleles in this region not annotated previously were revealed (see: GenBank, accession numbers KJ663713- KJ663725), making the general picture of genetic diversity even more complex. The rich deposit of polymorphic genes potentially influencing susceptibility to, and severity of, TB infection, as well as the potential contributions of both polymorphism and expression regulation of other genes in the region, justified further narrowing the interval by genetic recombination.

### Fine genetic mapping: round 2

**Fig 3:**
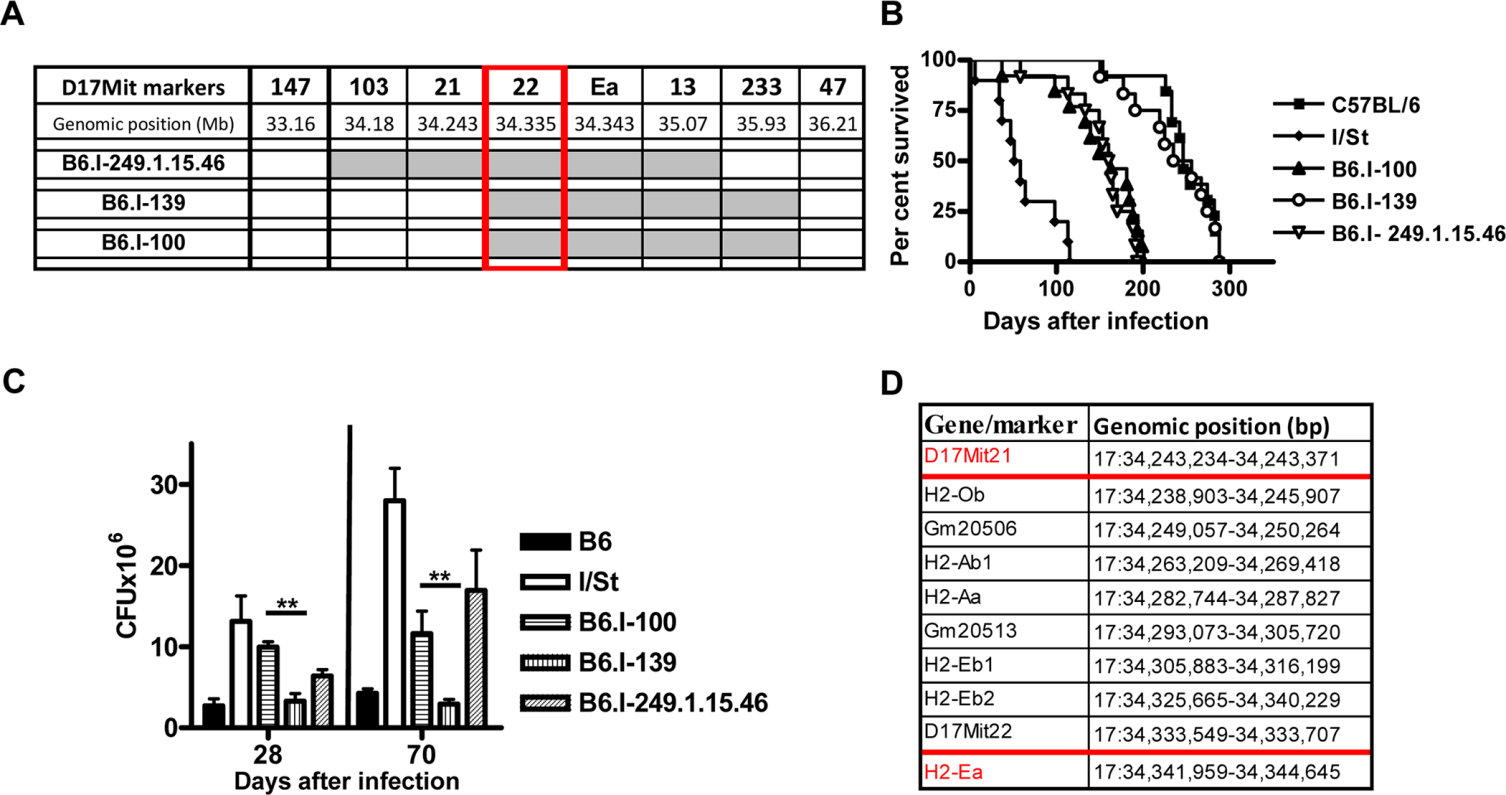
Genotypes and TB phenotypes of new recombinant mouse strains B6.I-100 and B6.I-139. **A–**The genome chart for B6.I-100 and B6.I-139 recombinant strains. Chromosome segments transferred from I/St onto B6 genetic background are shown in grey. **B–**survival curves of B6.I-100, B6.I-139 and parental strains mice after aerosol challenge with ~500 CFU of *M*. *tuberculosis*. MST ± SEM (days): B6 = 249 ± 10; I/St = 63 ± 11; B6.I-100 = 152 ± 13; B6.I-139 = 233 ± 14; B6.I-249.1.15.46 = 153 ± 11. Recombinant strains were tested in 3–5 independent experiments (total N = 20–40 males). Summary of 3 experiments is displayed (Kaplan-Meier survival analysis). **C–**CFU counts in infected lungs at days 28 and 70 post-challenge (4 mice per group, **P < 0.05, ANOVA). The representative results of one out of 2 independent experiments are present. **D–**genes and their positions in the D17Mit21 – *H2-Ea* interval according to Ensembl. Red borders–location of the candidate gene.

To search for new recombination events inside the region 33, 77–34, 34 Mb, we performed several crosses between novel recombinant and B6 mice. In particular, the F2 progeny of (B6.I-249.1.15 x B6) F1 mice was used to develop a new set of congenic strains. In two new recombinant strains, B6.I-249.1.15.100 (hereafter–B6.I-100) and B6.I-249.1.15.139 (hereafter–B6.I-139), standard genotyping identified the point of recombination between markers D17Mit21 and D17Mit22 (Fig 3A). Surprisingly, these strains demonstrated sharply contrasting TB phenotypes (Fig 3B). After aerosol challenge, B6.I-139 mice did not differ by survival time from parental B6 mice (mean survival time (MST) = 238.9 ± 13.41 and 249 ± 10.21 days, respectively, *P* > 0.5). B6.I-100 mice did not differ from the B6.I -249.1.15.46 strain (MST 152 ± 13.3 and 153 ± 10.97 days, respectively, *P* > 0.5), but did differ significantly from the B6.I-139 strain (*P* < 0.001). Phenotypic differences were confirmed by evaluation of cachexia dynamics (S2 Fig), and by assessment of mycobacterial loads in the lungs at weeks 4 and 10 post-challenge (Fig 3C). Annotation in the http://www.ensembl.org database provides the length of 98, 588bp for the genomic region between D17Mit21 and D17Mit22, which contains only 5 protein-coding genes, 2 lincRNA genes and no genes for micro-RNAs (Fig 3D).

### B6.I-100 and B6.I-139 mice carry different allelic variants of the *H2-Ab1* gene

**Fig 4:**
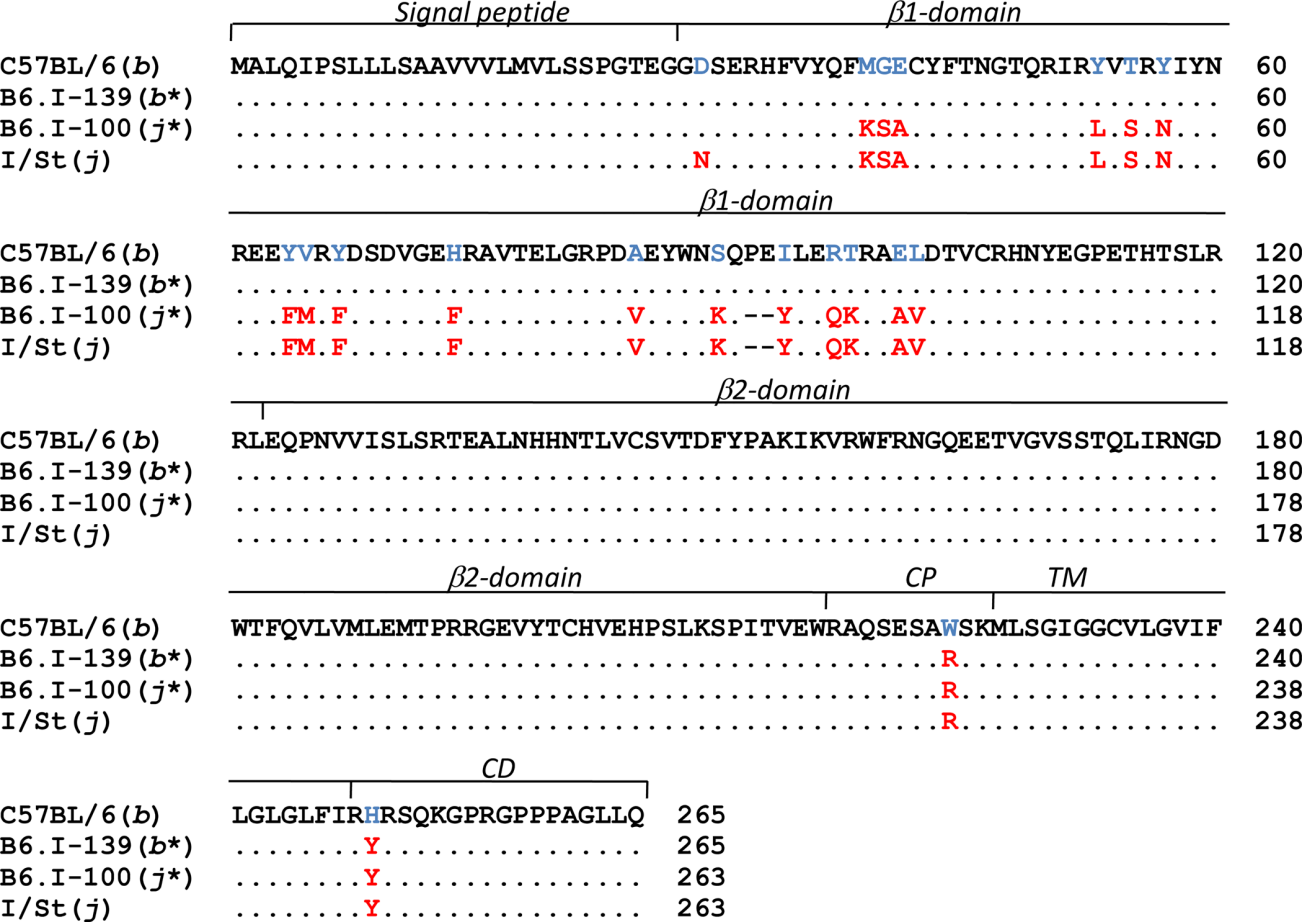
The differences in H2-Ab1 AA sequences between B6.I-100 and B6.I-139 mice. Protein structure alignment of H2-Ab1molecules. Gene annotations are from UniProt Domain structure http://www.uniprot.org: 1–27 –signal peptide; 28–122 - β1 polymorphic domain; 123–216 - β2 conservative domain; 217–226 –connecting peptide (CP); 227–247 –transmembrane domain (TM); 248–265 –cytoplasmic domain (CD). H2^b –^H2^j^ AA substitutions are highlighted.

The chromosomal segment sufficient to determine the contrasting TB phenotypes appeared to be very small, and we identified genetic material inside the segment by gene sequencing. Both strains carried the *b* allele of *H2-Ob* and the *j* allele of *H2-Aa*, but differed at the *H2-Ab1* gene (Fig 4). The *H2-Ab1* gene in both strains originated from recombination events between *b* and *j* haplotypes, but the crossing-over occurred at different sites. In B6.I-139 mice, the whole polymorphic part of the *H2-Ab1* gene encoding the extracellular functional domain of the molecule was of the *H2* ^b^ origin: only substitutions W222R in the connecting peptide and H249Y in the cytoplasmic domain were inherited from the *H2* ^j^ haplotype. In contrast, in B6.I-100 mice this polymorphic part of the *H2-Ab1* gene was identical with that of the *H2* ^j^ haplotype, except for a single substitution N29D (Fig 4). As far as both recombination events occurred in the translated part of the gene, we assume that the promoter region of B6 origin was identical for both strains and played no role in infection response. The fact that B6.I-139 mice displayed the resistant phenotype similar to parental B6 mice suggests that two AA substitutions of I/St origin in the connecting peptide and the cytoplasmic domain are not major players in TB susceptibility. Analogously, the presence of the *H2* ^b^-encoded aspartic acid in the *H2-Ab1* of the B6.I-100 strain is unlikely to influence the level of TB susceptibility, since B6-I.100 mice display a phenotype identical to that of B6.I-249.1.15.46 mice, whose entire *H2-Ab1* gene was inherited from I/St mice. Taken together, these results demonstrate that the differences in TB susceptibility/severity between these two recombinant mouse strains were determined by allelic polymorphisms in a single β1 domain of the H2-Aβ molecule.

Thus, independent recombination events within a single gene created genetic variation sufficient to markedly alter the response to TB infection. The newly identified *H2-Ab1* alleles were designated as j* in the B6.I-100 strain and b* in the B6.I-139 strain.

### Parameters of infection regulated by the *H2-Ab1* alleles

**Fig 5:**
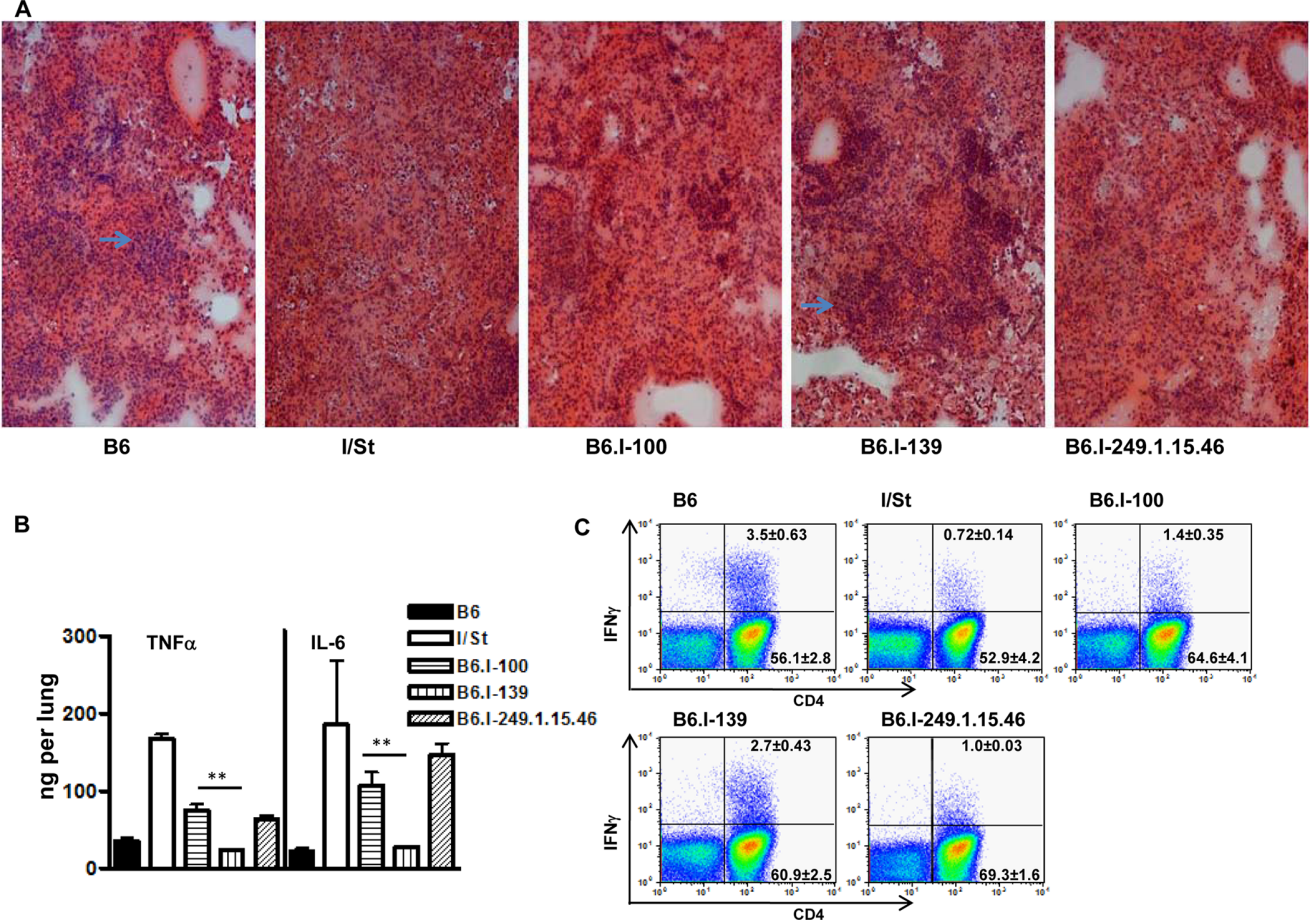
Possession of the *H2-Ab1* ^j^-like alleles results in a more severe TB infectious course. **A–**Representative tuberculous lung lesions at day 35 post-infection. Hematoxylin and eosin staining, magnification x100. Arrows show granulomatous structures. **B**–TNF-α and IL-6 production in infected lungs at day 60 post-challenge. Whole-lung homogenates from individual mice (3 per group) were assessed in the ELISA format. The results are expressed as mean ± SD from two independent experiments (total N = 6), *P* < 0.05 for B6.I-100 and B6.I-139, ANOVA. **C–**The number of lung IFN-γ-producing CD4+ T cells was assessed by intracellular staining for IFN-γ at 35 days post-challenge. After culturing with mycobacterial sonicate, the lung cell population gated for CD3 expression was analyzed as displayed. Results of one of two similar experiments (total N = 6) are shown, with statistics for 3 individual mice per group provided in quadrants. In controls (cells from normal mice with mycobacterial sonicate, or cells from infected mice without antigen in culture) the per cent of IFN-γ-producing lung CD4+ T cells never exceed 0.1. *P* < 0.05 for B6.I-100 and B6.I-139, ANOVA.

We performed fine genetic mapping using the most integrative TB characteristics–survival curves, mycobacterial multiplication in the lungs, and body weight loss. Differences in the regulation of lung tissue inflammation after infection in B6 and I/St mice are of critical importance for TB pathogenesis [46, 47]. To characterize the influence of the *H2-Ab1* polymorphism on TB-induced inflammation, we compared lung pathology 35 days post-infection in mice of both parental and new recombinant strains. As shown in Fig 5A, in B6 and B6.I-139 mice, lung pathology was represented by granulomatous areas well-delimited from the breathing tissue, whereas I/St and B6.I-100 mice developed diffuse TB pneumonia that was more severe in I/St mice.

In good agreement with the histological results, the levels of key Type 1 inflammatory cytokines, IL-6 and TNF-α, after TB challenge were significantly lower in the lungs of resistant B6 and B6.I-139 mice compared to susceptible I/St and B6.I-100 mice (Fig 5B). No difference in the levels of the TB-irrelevant Type 2 cytokine IL-5 between all four strains was found. Importantly, production of the key TB-protective Type 1 cytokine, IFN-γ, by CD4^+^ T-lymphocytes isolated from the lungs of infected mice and stimulated in vitro with a mixture of mycobacterial antigens followed the *H2-Ab1*-determined pattern. As shown in Fig 5C, the 5-fold difference in the numbers of IFN-γ-producing CD4^+^ T cells between parental B6 and I/St strains was reduced to a 2-fold difference between B6.I-139 and B6.I-100 mice but remained highly significant (*P* < 0.01). Calculation of the total numbers of IFN-γ-producing CD4 cells per lung provided the results consistent with the percentile evaluation (S2 Table). These results establish connections between anti-TB protective immune responses, CD4^+^ T-cell function and allelic heterogeneity of the classical Class II antigen-presenting molecule, providing a mechanistic explanation for the differences in the severity of disease determined by a single *MHC* gene.

### CD4^+^ T cell response to mycobacterial antigens develops in the context of the H2-A molecule

IFN-γ production by the CD4^+^ T cell in response to MTB is generally considered the major mechanism of host defense [48]. TB-resistant B6 mice express only one MHC Class II molecule - H2-A^b^ on their antigen-presenting cells (APC), whereas I/St mice express both MHC Class II molecules - H2-A^j^ and H2-E^j^. *A priori* it was impossible to judge whether the defect in TB defense in mice bearing the *H2* ^j^ haplotype was determined by sub-optimal antigen presentation by the H2-A^j^ compared to the H2-A^b^ molecule, or by the parallel presentation of mycobacterial antigens by two Class II molecules which somehow interfered with the development of protective immunity. To resolve this issue, we assessed the presentation of mycobacterial antigens by the APC derived from mice with different Class II allelic composition.

**Fig 6:**
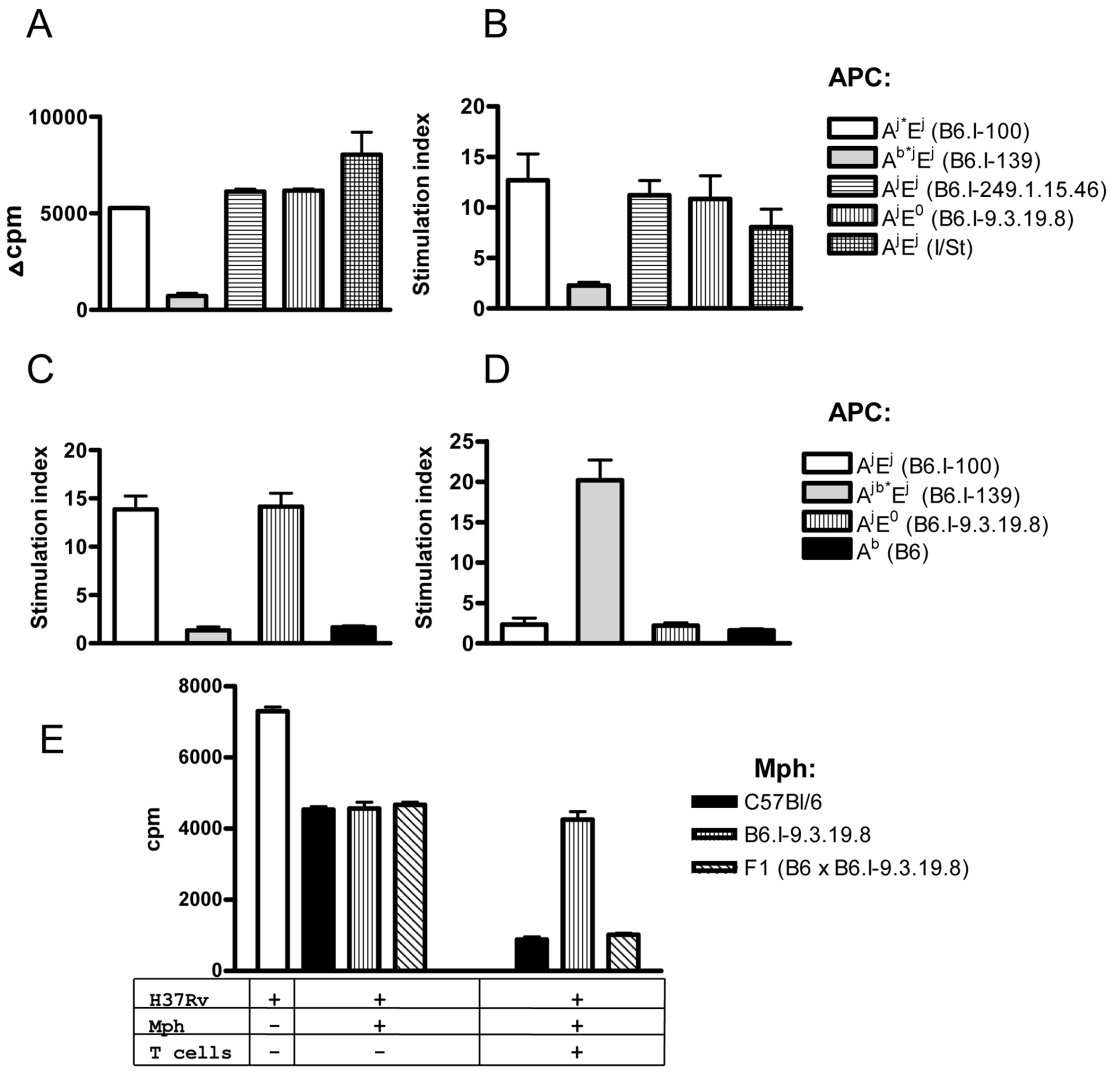
CD4^+^ T cells recognize mycobacterial antigens in the context of H2-A molecule. Mycobacterial antigens were presented by APC expressing different gene combinations and alleles of Class II molecules (see legend) to: (A) polyclonal I/St T cell line, or to highly purified immune lymph node CD4+ T cells (pooled from 2–3 mice) from I/St (B), B6.I-100 (C) and B6.I.139 mice (D). Representative data from one out of two similar independent experiments are presented, results are expressed as mean ± SEM of triplicate cultures. Y-axis: ***Δ cpm*** (counts per minute) = mean ***cpm*** of antigen-stimulated wells—the mean ***cpm*** of non-stimulated wells. ***Stimulation index*** (SI) mean ***cpm*** of antigen-stimulated wells/ mean ***cpm*** of non-stimulated wells. (E)–CD4^+^ T-cells from (B6 x B6.I-9.3.19.8) F1 mice stimulate bacteriostatic activity of B6 and F1, but not of B6.I-9.3.19.8 macrophages (mph). Results are present as [^3^H]-uracil uptake from one out of two experiments provided similar results (CPM ± SEM for triplicates, *P* < 0.001, ANOVA). See Materials and Methods for details.

A mycobacteria-specific CD4^+^ T cell line derived from I/St mice [49] readily proliferated in the presence of mycobacterial antigens if the APC were derived from mice expressing the H2-A^j^ molecule, even if the H2-E molecule was not expressed (Fig 6A). Moreover, the presence or absence of H2-E did not change the level of response, suggesting that the H2-A molecule presents mycobacterial antigens to the vast majority of T-cell clones. To prove that the H2-E-recognizing T-cell clones have not been lost due to repeated stimulation during T-cell line development, we repeated the experiment with highly purified CD4^+^ T cells from TB-immune lymph nodes of I/St mice and obtained similar results (Fig 6B).

Recombinant B6.I-100 and B6.I-139 mice express the H2-E molecule due to the presence of the H2-E^j^ α-chain. These mice possess identical H2-A^j^ α-chains but differ in their H2-A^j^ β-chains (Fig 4). To determine in which context mycobacterial antigens were presented to T cells by newly originated *H2* haplotypes and to evaluate the efficacy of antigen presentation by recombinant H2-Aβ chains, we assessed the response of T-cells from recombinant mice in the presence of different APC. As shown in Fig 6C and 6D, B6.I-100 and B6.I-139 T cells recognized mycobacterial antigens in the context of the H2-A, but not the H2-E, molecule. Interestingly, B6.I.100 T-cells did not distinguish fully syngenic A^j*^ and progenitor A^j^, whereas B6.I-139 T cells recognized only A^b*^ β-chain, most likely because the hybrid H2-Aβ^b^/Aα^j^ molecule was formed. Taken together, these results indicate that the H2-A molecule plays a pivotal role in the presentation of mycobacterial antigens and the generation of TB-specific CD4^+^ T cell responses. The A^j^ and A^j*^ allelic variants are not intrinsically defective in the antigen-presenting function and elicit a level of T cell proliferation in response to soluble mycobacterial antigens similar to the A^b^ and A^b*^ alleles.

These observations provided an opportunity to functionally test whether or not the result of T cell interaction with infected macrophages depends upon *H2-A* alleles. To this end, we performed co-culture experiments with macrophages from B6 and congenic B6.I-9.3.19.8 (see Fig 1) mice and immune effector CD4 T-cells obtained from (B6 x B6.I-9.3.19.8) F1 mice, which are able to interact with both allelic forms of H2-A. The H2-E-negative strain B6.I-9.3.19.8 was used instead of B6.I-100 to further exclude possible influence of the H2-E expression. As shown in Fig 6E, T-cells profoundly increased the ability of B6 and F1 peritoneal macrophages to inhibit mycobacterial growth, whereas only marginal effect was seen in B6.I-9.3.19.8 macrophages, suggesting that recognition of “protective” H2-A^b^ vs. “non-protective” H2-A^j^ molecules by CD4^+^ T-cells leads to profound differences in macrophage activation. Remarkably, moderate capacity to inhibit mycobacterial growth in the absence of T-cells was similar in all macrophages, regardless their genetic origin. This is in full agreement with theoretical expectations: Class II alleles do not regulate the level of innate protective response. These results provide additional independent conformation in support of the conclusion that the *H2-A* allelic variation is sufficient to determine prominent variations in acquired anti-mycobacterial immunity. In contrast with experiments on genetic restriction of antigen-specific response described above, reciprocal functional experiment (activation of F1 macrophages by *H2-A* ^*b*^ and *H2-A* ^*j*^ T-cells) would not be informative, since allogenic Class II recognition provides unpredictable effects.

### Molecular modeling

A BLAST search in the Protein Data Bank (PDB) for α- and β-chains of the H2-A^j^ molecule revealed the highest score of sequence similarity (88% and 85% identity, respectively) with the protein 2P24 [50], with deletions or insertions lacking. Comparison between H2-A^j^ and H2-A^b^ (PDB id 1MUJ) provided sequence identity of 93% and 89% for α- and β-chains, respectively, with the 2-AA deletion (P65 and E66) in the former allele. Two molecular models of the H2-A^j^ protein based upon atomic coordinates of 1MUJ and 2P24 provided a high level of similarity around the deletion point (S1 Fig), which justified the use of the 1MUJ model for further comparisons.

**Fig 7:**
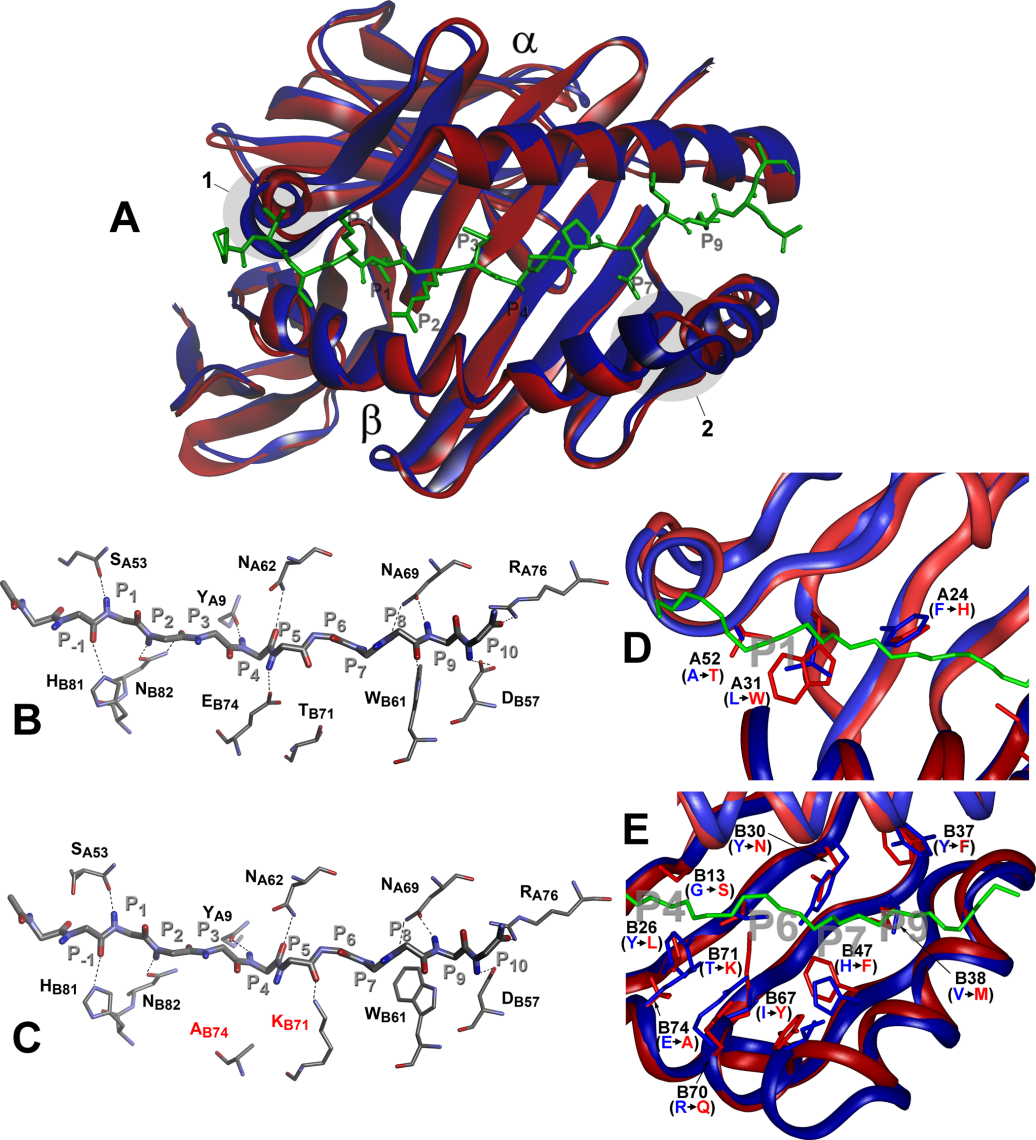
Molecular model of H2-A^j^ molecule in comparison to H2-A^b^. Top view of structural overlay of the peptide-binding domains of H2-A^b^ (blue) and H2-A^j^ (red) alleles, bound to CLIP peptide (green). α- alpha and β–beta chains. 1 –α- subunit 3_10_ helix, 2- β subunit region with two AA (P65E66) deletions in j-haplotype **(A)**. Comparison of the H-bond network between H2-A^b^ (**B**) and H2-A^j^ (**C**) molecules containing CLIP peptide backbone (P-1-P10). MHC Class II conserved residues that contribute to the peptide- MHC hydrogen-bonding network are shown in stick representation. Dashed lines indicate conservative hydrogen bonds with the exception of Ab74 and Kb71 (marked red) in the H2-A^j^ molecule. **D** -Comparison of pocket structures of the MHC- binding groove between H2-A^b^ (blue) and H2-A^j^ (red). The CLIP peptide backbone is shown in green, P1, P4, P6, P7 and P9 pockets in grey. AA substitutions in the α-chain contribute mostly to the differences in the P1 structure, AA substitutions in the β-chain determine differences in P4, P6, P7 and P9 pockets (**E**). Potentially most important substitutions are marked and their side chains shown.

Comparison between the H2-A^j^ and H2-A^b^ molecules suggests that the most prominent structural dissimilarities occur in two protein backbone regions: the 3_10_ helical fragment of the α-chain and the P65E66 deletion in the H2-Aβ^j^ chain (Fig 7A). These deviations are not unique and are present in other *H2* haplotypes (reviewed in Ref [51] and displayed in S3 Fig). Analysis of the hydrogen bond network between H-2A Class II molecules and, as a model, invariant CLIP peptide stabilizing the complex before an antigenic peptide is loaded, demonstrated that the conserved H-bond interactions and the total number of H-bonds are identical for the H2-A^b^ and H2-A^j^ products despite two b → j substitutions, T71K and E74A in the Aβ-chain (Fig 7B and 7C). The Aβ-position E74 is highly conserved among all known mouse *H2-A* haplotypes (S4 Fig) and the majority of human HLA-DQ molecules [52]. In the H2-A^j^ molecule, the A74-provided H-bond is lacking; however, it might be functionally substituted by the H-bond from the Aβ K71.

However, prominent differences in the structure and size of the peptide-anchoring pockets between the two allelic forms of the H2-A molecule were observed. Structural data for the peptide-anchoring pockets were either available from the 3D structure of the H2-A^b^ [53] or deduced for the H2-A^j^ from our model. AA substitutions distinguishing the H2-A^j^ protein from the prototypic H2-A^b^ should have profoundly changed the structure of the peptide-binding groove in binding pockets P1, P4, P6, P7 and P9. Due to the substitution L31W and A52T in the α-chain, the volume of the H2-A^j^ P1 pocket should be appreciably smaller compared to that of H2-A^b^ (Fig 7D) and, therefore, may accept smaller side chains capable of forming a hydrogen bond with the Aβ T52 hydroxyl group. Changes in other binding pockets are due to substitutions in the β-chain (Fig 7E). The differences between H2-A^j^ and H2-A^b^ in the P4 binding pocket include substitutions Y26L, T28S, G13S and the most prominent one—E74A, allowing occupation of the pocket by a neutral residue in H2-A^j^ instead of a positively charged residue in H2-A^b^. Changes in the P6, (substitutions Y30N and T71K), make this pocket in the H2-A^j^ molecule more permissive for negatively charged residues. Due to the H47F substitution, the pocket P7 is neutral in H2-A^j^ but positively charged in H2-A^b^, while a minor substitution Y37F makes pocket P9 more permissive for lipophilic residues. We consider the most important differences between H2-A^j^ and H2-A^b^ to result from a unique substitution Q61K in the α-chain and substitution R70Q in the β-chain, since these substitutions alter the polarity of interactions between two chains (S5 Fig). Two positively charged residues occupying opposite positions in the hybrid H2-A (α^j^+β^b*^) (S5 Fig) could well affect the Class II-TCR interactions.

## Discussion

An extremely high level of genetic polymorphism in the *H2* chromosomal region and its unique saturation with immunity-relevant genes complicate the identification of allelic variants in a particular gene influencing the disease severity and outcome. This is particularly true for the classical Class I and II genes, for which genetic silencing approaches are hardly applicable since they lead to a severe abrogation of overall functions of acquired immunity. It took us about 7 years to develop the panel of more than 40 *H2*-recombinant inbred strains on the B6 genetic background sufficient for identification of the *H2-Ab1* gene as the TB severity determinant using the forward genetic approach. The key point was establishing the fact that in the B6.I-100 and B6.I-139 strains distinct recombination breakpoints between the *H2* ^j^ and *H2* ^b^ haplotypes were located within the same *H2-Ab1* gene. This resulted in a *H2-Ab1* ^b^-like allele in TB-resistant B6.I-139 and in a *H2-Ab1* ^j^-like allele in TB-susceptible B6.I-100 mice, which, being compared with previously characterized phenotypes and genotypes in other strains from the panel, identified the *H2-Ab1* as the gene underlining the Chromosome 17 TB-controlling QTL. This is the first direct demonstration of the differences in TB infection susceptibility/severity depending on allelic polymorphisms in a single Class II *MHC* gene. Associations of TB susceptibility/severity with the MHC polymorphic haplotypes have been previously reported both in humans [27–30] and mice [31–35], but direct evidence that the alleles of *H2-A1* or its human orthologous gene *HLA-DQ* differentially regulate TB control by the host was lacking. Importantly, in our system both allelic variants apparently retain normal functional activity and are not defective in mycobacterial antigen presentation/recognition (Fig 6). This may reflect the situation which exists in natural populations: fine genetic differences may lead to pronounced shifts in adaptively important responses providing the subject for slowly operating natural selection on quantitative basis, whereas loss-of-function mutations in key immunologically active genes are eliminated rapidly. Allelic differences in a single *H2-A1* gene influenced all major phenotypes characterizing severity of TB infection (bacterial loads in affected organ, histopathology, cachexia, survival time post-challenge), suggesting that *H2-Ab1* is one of major players in the TB control in mice. Naturally, this does not exclude the presence of other genes within an extended *H2* region involved in TB control, but their possible contribution is likely to be weaker.

The established panel of recombinant mouse strains may serve a useful tool for dissecting genetic control of susceptibility/severity in other models of TB and in other experimental infections. Indeed, one TB-susceptibility QTL, *Sst5*, has been mapped to the *H2* region in the B6 –C3HeB/FeJ mouse strain combination [21]. An extended *H2* region contains QTL involved in genetic control of susceptibility/severity of several protozoal and metazoal pathogens: *Lmr1* for *Leishmania major* [54], *Char3* for *Plasmodium chabaudi* [55], *Belr1* for *Plasmodium berghei* [56], *Tir1* for *Trypanosoma congolense* [57] and *Sm2* for *Schistosoma mansoni* [58]. It is very unlikely that co-localization of all these QTL is coincidental, and our new panel of mouse strains may shed light on the architecture of the *H2*-driven genetics of host-parasite interactions in these other disease models.

Regarding the molecular mechanisms underlining differences in TB susceptibility in the absence of overt gene dysfunctions, several possibilities are being considered. The most evident is differences in mycobacterial antigenic peptide repertoire presented to T-cells by structurally different Class II molecules. Alignment of the AA sequence of the polymorphic *H2-Ab1* ^j^ domain with annotated *H2* haplotypes (S4 Fig) demonstrates that it shares the common deletion P65-E66 with haplotypes *k*, *g7*, *u*, *s*, *f*, *s2*, retains conserved AA residues at positions determining the basic 3D structure of the protein, and is not unique with this regard. However, there are non-conservative and potentially important substitutions located in four β-strands and in the α-helix that form the peptide-binding groove. Molecular modeling predicts that the motif for peptide binding should differ between H2-A^j^ and H2-A^b^ due to substitutions in the pockets P4, P6, P7 and P9 (Fig 7). However, at present no information is available concerning sets of mycobacterial peptides providing more *vs*. less protective anti-TB responses.

We also considered the level and stability of the cell surface expression of the H2-A allelic forms as a factor potentially influencing the level and quality of T-cell activation. Since antibodies reacting with the H2-A^b^ and H2-A^j^ molecules with equal affinity are lacking, a direct quantitative comparison of expression levels was impossible. Thus we applied an antibody dilution approach described earlier [59, 60] and found no differences in the expression levels for the H2-A molecule between B6, B6.I-100 and B6.I-139 mice (S6 Fig), most likely excluding this explanation.

Yet another possible reason for the differences in anti-mycobacterial immunity between the carriers of *H2-Ab1* ^j^ and *H2-Ab1* ^b^ alleles could be selection of CD4 T cells in thymus and/or their maintenance in the periphery. Our preliminary studies demonstrated a significant difference in the CD4: CD8 ratio between B6 and I/St mice, as well as between some of the novel recombinant mice. More data and, possibly, new recombinant mouse strains expressing no H2-E molecule will be needed to precisely evaluate the importance of this *MHC-*dependent pathway of immune response regulation.

## Materials and Methods

Mice of inbred strains I/StSnEgYCit (abbreviation I/St, *H2* ^j^) and C57BL/6JCit (abbreviation B6, *H2* ^b^) were bred and maintained under conventional, non-SPF conditions at the Animal Facilities of the Central Institute for Tuberculosis (CIT, Moscow, Russia) in accordance with the guidelines from the Russian Ministry of Health # 755, and under the NIH Office of Laboratory Animal Welfare (OLAW) Assurance #A5502-11.

The B6.I panel of *MHC-*congenic strains (fragments of the *H2* ^j^ haplotype transferred onto B6 genetic background; totally, more than 40 strains) was established using the classical cross–backcross–intercross protocol [61]. Selection of backcross progeny carrying the *H2* ^j^ haplotype of the I/St origin and its inter-*MHC* recombinant derivatives with the *H2* ^b^ haplotype was performed in each backcross generation. Genotypes of the simple sequence length polymorphisms in the region under study (SSLPs *D17Mit*, www.jax.org) were determined using PCR of isolated tail DNA samples (Wizard Genomic DNA Purification Kit, Promega, USA) followed by product separation on 4–6% agarose gels. The following markers were used: D17Mit13, 16, 21, 22, 28, 47, 49, 57, 81, 82, 103, 133, 143, 147, 152, 175, 177, 233. Two SNP markers, rs13482956 and rs50366565, were genotyped by PCR followed by enzymatic digestion of PCR products with FspBI and Hin1II, respectively (Thermo Scientific, Lithuania).

All carriers of novel *MHC-*region allelic variants were serially backcrossed on B6 parental mice. After generation N = 10–14, homozygous animals were obtained by sib mating and further maintained by brother-sister mating. Water and food were provided *ad libitum*. Mice of 8-12wk of age at the beginning of experiments were used. All experimental procedures were approved by the CIT Animal Care Committee (IACUC protocols #2, 7, 8, 11 approved on March 6, 2013).

### Infection and major phenotypes

To evaluate severity of the disease, mice were infected with ~5 x 10^2^ colony-forming units (CFU) of standard virulent *M*. *tuberculosis* strain H37RV (sub-strain Pasteur) using an Inhalation Exposure System (Glas-Col, Terre Haute, IN) exactly as described earlier [49]. Mortality was monitored daily starting at week 5 post-infection. To assess CFU counts, lungs from individual mice were homogenized in 2.0 ml of sterile saline, and 10-fold serial dilutions were plated on Dubos agar (Difco) and incubated at 37°C for 20–22 days. Pathology of the lung tissue was assessed as described [50]. Briefly, mice were euthanized by a thiopental (Biochemie GmbH, Vienna, Austria) overdose. Lung tissue (the middle right lobe) was frozen in a –60°C to –20°C temperature gradient in an electronic Cryotome (ThermoShandon, UK), 6–8μm-thick sections were cut across the widest area of the lobe, fixed with acetone, stained with hematoxylin-eosin and mounted.

### Stimulation/rest protocol for mycobacteria-specific T-cell lines and proliferation assays

To prepare T-cell lines, cells from the popliteal lymph nodes of I/St and B6 mice, immunized into rear footpads with 10μg/mouse of mycobacterial sonicate mixed 1:1 with incomplete Freund’s adjuvant, were cultured as described previously [62]. Briefly, 2 x 10^6^/ml immune cells isolated on day 21 post-immunization were cultured in 24-well plates (Costar, Netherlands) in RPMI-1640 containing 10% FCS, 10 mM HEPES, 4 mM L-glutamine, 5 x 10^−5^ M 2-ME, vitamins, pyruvate, non-essential amino acids and antibiotics (all components–HiClone, Logan, UT, USA) for 14–16 days in the presence of 10μg/ml mycobacterial sonicate. Live immune cells (>93% viability by trypan blue exclusion) were isolated by centrifugation at 2500 g for 20 min at 20°C, on the Lympholyte M gradient (Cedarlane Labs, Ontario, Canada), washed twice and counted. The next stimulation cycle was accomplished by co-culturing 2 x 10^5^ isolated cells with mitomycin C-treated 1.5 x 10^6^ splenic APC in the presence of sonicate for another 14–16 days. These cycles were repeated 4 times and resulted in stable antigen-specific CD4^+^ (>99% purity by flow cytometry) T-cell lines. To obtain fresh immune CD4^+^ T cells, at day 21 following immunization lymph node cells were purified by negative selection using magnetic beads (CD4^+^ T-cell Isolation kit II, Miltenyi Biotec) according to the manufacturer’s recommendations.

To assess antigen-specific proliferation, either 10^5^ purified CD4^+^ T cells, or 10^4^ T-line cells were co-cultured with 2 x 10^5^ mitomycin C-treated splenic APC in a 96-well flat-bottom plate (Costar), at 37°C, 5% CO_2_, in supplemented RPMI-1640 containing 10 μg/ml of H37Rv sonicate. Non-stimulated wells served as controls. Triplicate cultures were pulsed with 0.5 μCi [^3^H]-thymidine for the last 18 h of a 40 h incubation. The label uptake was measured in a liquid scintillation counter (Wallac, Finland) after harvesting the well’s contents onto fiberglass filters using a semi-automatic cell harvester (Scatron, Norway).

### Stimulation of macrophage bacteriostatic effect by T-cells

Peritoneal macrophages were obtained after stimulation with peptone as described previously [63]. 50 x 10^3^ macrophages per well of 96-well plates in RPMI-1640 supplemented with 2% FCS and containing no antibiotics were infected with *M*. *tuberculosis* H37Rv at MOI 5:1 for 1.5 h. CD4^+^ T cells (~97% purity) were obtained from spleens of (B6 x B6.I-9.3.19.8) F1 mice at day 21 after i. v. infection with 5 x 10^5^ CFU of *M*. *tuberculosis* H37Rv using magnetic separation (see above). T cells were added to infected macrophages at 1:1 ratio, and co-cultures kept for 72 h at 37°C in CO_2_ incubator. To assess mycobacterial viability, [^3^H]-uracil label was added for last 18 h of incubation, and the uptake assessed exactly as described in [63]. This method provides >99% correlation with CFU counting [63].

### Cell preparations and flow cytometry

Infected B6 and I/St mice were euthanized by thiopental overdose, and lung cell suspensions were prepared using the methods described earlier [64]. Briefly, blood was washed out by repeated broncho-alveolar lavage with 0.02% EDTA-PBS with antibiotics, lungs removed, sliced into 1–2 mm^3^ pieces and incubated at 37°C for 90 min in supplemented RPMI-1640 containing 200 U/ml collagenase and 50 U/ml DNase-I (Sigma, MO). Single cell suspensions obtained by vigorous pipetting were washed twice in HBSS containing 2% FCS and antibiotics. Suspensions of spleen and lymph node cells were obtained using routine procedures. Cells were incubated 5 min at 4°C with an anti-CD16/CD32 mAb (BD Biosciences) for blocking Fc-receptors and stained with FITC-anti-CD3, APC-anti-CD8 and PerCP-anti-CD4 antibodies (BD Biosciences).

For intracellular IFN-γ staining, 1.5 × 10^6^ cells were cultured in 24-well plates in the presence of 10 μg/ml mycobacterial sonicate for 48 h; GolgiPlug block (1 μl/ml; BD Biosciences) was added for the last 10 hours. Cells were then stained with anti-IFN-γ mAb XMG1.2 (BD Biosciences) using the Cytofix/Cytoperm kit (BD Biosciences). The expression of the Н2-Еα molecules on cell surface, discriminating *Н2* ^b^ (Н2-Еα-negative) and *Н2* ^j^ (Н2-Еα-positive) haplotypes was assessed using the PE-14-4-4S mAb (BD Biosciences) Cells were analyzed on BD Biosciences FACSCalibur flow cytometer using CellQuest and FlowJo software.

The levels of cytokines in the lung tissue was measured individually in infected animals using whole-lung homogenates in 2 ml of sterile saline stored at –70°С before assessment. After thawing, debris was removed from the samples by centrifugation at 800 g, and cytokine levels in supernatants were assessed in an ELISA format using mouse OptEIA TNF-α Set, OptEIA IL-6 Set and OptEIA IL-5 Set (BD Biosciences) and mouse INF-γ Set (Biolegend) according to the manufacturer’s instructions.

### RNA purification and cloning of candidate genes

RNA was extracted from spleens using the SV Total RNA Isolation System (Promega, USA) and treated with DNase I (AMPD1, Sigma). Complementary DNA (cDNA) was synthesized with oligo-dT18 primers (Thermo Scientific, Lithuania) and M-MLV reverse transcriptase (Promega, USA). Primer sequences for cloning were obtained from the Ensembl database (version GRCm38.p2) for the C57BL/6 strain. 5'(forward) primers ended at the start codon (ATG); reverse primers started at the (TGA) stop codon. Coding DNA was amplified with Advantage GC Genomic LA Polymerase (Clontech, USA), PCR products were purified by gel extraction with Cleanup Mini Set (Evrogen,Russia) and cloned into the PCR-Script Amp Cloning vector using the PCR-Script Amp Cloning Kit (Stratagene, USA) or in pAL-TA (Evrogen, Russia) with preliminary 3 cycles of amplification of PCR products with Taq polymerase (*H*elicon, Russia). The 4–6 positive clones were sequenced for each gene. Nucleotide sequences have been submitted to the GenBank (http://www.ncbi.nlm.hih.gov/genbank) under accession numbers KJ650201-KJ650234, KJ663-713-KJ663725.

Molecular modeling was performed using an Octane2 workstation (Silicon Graphics, USA) equipped with the programs Insight II/Discover (Accelrys, USA). Atomic coordinates of the mouse Class II MHC H2-A^u^ MBP125-135 (PDB id 2P24) [50] and Class II MHC H2-A^b^ in complex with the human CLIP peptide (PDB id 1MUJ) [54] were used for homology modeling. AA substitutions were deduced using the Biopolymer program. In order to minimize inter-atomic clashes, all individual AA conformations were chosen automatically, using the criteria of the lowest energy. To deduce the structure of the H2-A^j^ molecule using the H2-A^b^ template, the deletion P65-E66 was introduced manually using the Biopolymer program. Atomic coordinates available from two models, 1MUJ and 2P24, for the Class II-CLIP structures were subjected to further refinement for the I-A^j^ using the Discover program and AMBER force field. Refinement stages included short energy minimization (the steepest descent algorithm), followed by 1 ps molecular dynamics simulations at 298K and by the final energy minimization (the conjugate gradient algorithm). Results were visualized using the Insight II and Discovery Studio software (Accelrys, USA).

### Statistical analysis

All analyses were done using Graphpad Prism version 4. Mortality was assessed using Kaplan-Meier survival analysis and the log-rank tests, CFU counts using Student’s *t*-test. *P* < 0.05 was considered statistically significant.

## Supporting Information

S1 Fig The dynamics of body weight loss by parental B6 and I/St and recombinant congenic mice after infection. The set of recombinant strains, which express susceptible phenotype, is displayed in red. N = 10–15 for each strain in the beginning of experiment, the dynamics of weight for each strain was normalized to zero at day 0.
(TIF)

S2 Fig Difference in the dynamics of body weight loss between B6.I-100 and B6.I-139 strains compare to ancestor strains. (TIF)

S3 Fig Molecular modeling of the β51–70 region. Superposition of two molecular models of the H2-A^j^ protein obtained by the homology modelling approach from atomic coordinates of 1MUJ (red) and 2P24 (purple). Fragments B51-B71 of the β-chain are shown. The sequence of H2-A^j^ differs from the 1MUJ (but not from 2P24) by deletion of residues 65 and 66.
(TIF)

S4 Fig Polymorphism of H2-A alleles of inbred mouse strains. Alignment of α1 and β1 polymorphic domains of the H2-A^j^ α- (**A**) and β- (**B**) chain with annotated haplotypes of inbred mouse strains (left column). Sequences were taken from IMGT (http://www.imtg.org) database. The features of secondary structure are given according to (Lefranc et al., Develop. Compar. Immun, 2005; 29: 917–938). Yellow arrows mark four beta strands, the red spiral is α-helix. Multiple sequence alignment was made using ClustalW2 program (Larkin et al., Bioinformatics 2007, 23: 2947–2948).
(TIF)

S5 Fig Models of the MHC binding groove for H2-A alleles. Solvent accessible surface representation of the MHC-binding groove in H2-A^b^ (**A**), H2-A^j^ (**B**) and hybrid H2-A^jb*^ (**C**) molecules. Molecular surfaces are colored according interpolated electrostatic potential. Heavy atoms of the CLIP peptide are shown as sticks colored grey (carbon), blue (nitrogen) and red (oxygen). AA residues in positions α61 and β70 may form a salt bridge and are potentially available for interaction with TCR.
(TIF)

S6 Fig Cell surface expression of the H2-A molecule in B6.I-100, B6.I-139 and ancestor mice. Spleen cells were stained with serial dilutions of the anti-I-A^p^ mAb (clone 7–16.7; BD Biosciences) and analyzed by flow cytometry.
(TIF)

S1 Table Generally relevant and specifically TB-related activity of polymorphic genes located in the identified *H2* segment. (DOC)

S2 Table TB-resistant mice contain more CD4^+^ mycobacteria-specific IFN-*γ*-producing T-cells in their lungs^#^. (DOCX)
