# Supplementary figures and images for: The QTL within the H2 Complex Involved in the Control of Tuberculosis Infection in Mice Is the Classical Class II H2-Ab1 Gene
Source: PLoS Genet. 2015 Nov 30;11(11):e1005672. doi: 10.1371/journal.pgen.1005672 (PMC4664271; doi:10.1371/journal.pgen.1005672)

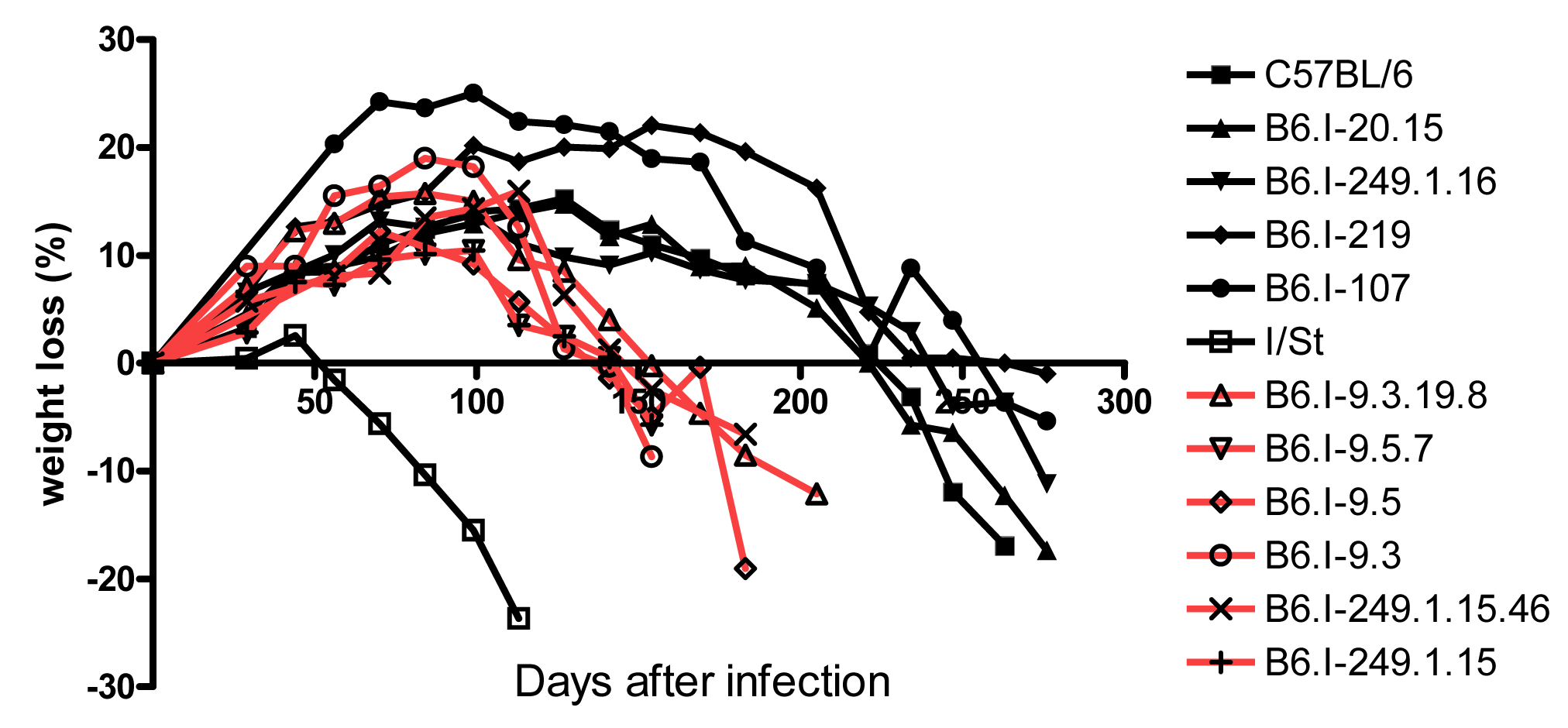

Supplement: S1 Fig — The set of recombinant strains, which express susceptible phenotype, is displayed in red. N = 10–15 for each strain in the beginning of experiment, the dynamics of weight for each strain was normalized to zero at day 0. (TIF) [file pgen.1005672.s001.tif]

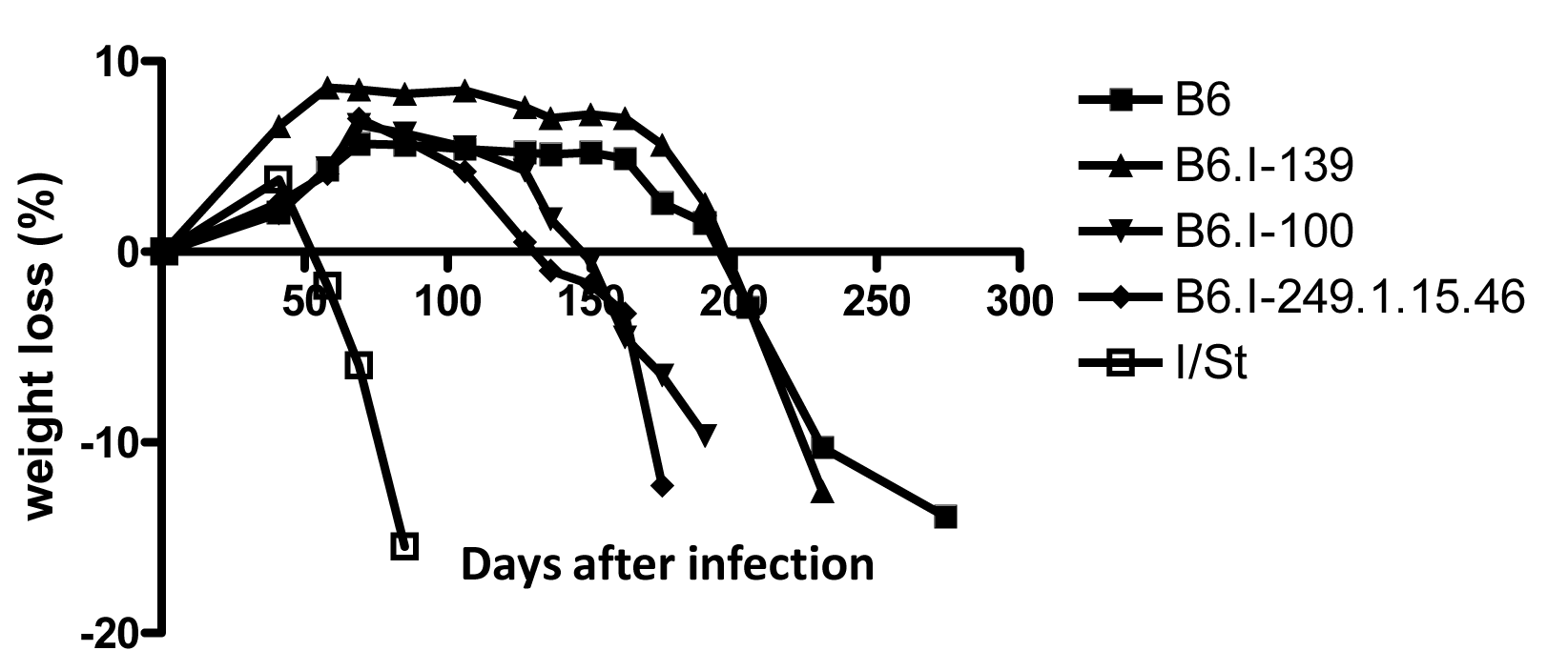

Supplement: S2 Fig — (TIF) [file pgen.1005672.s002.tif]

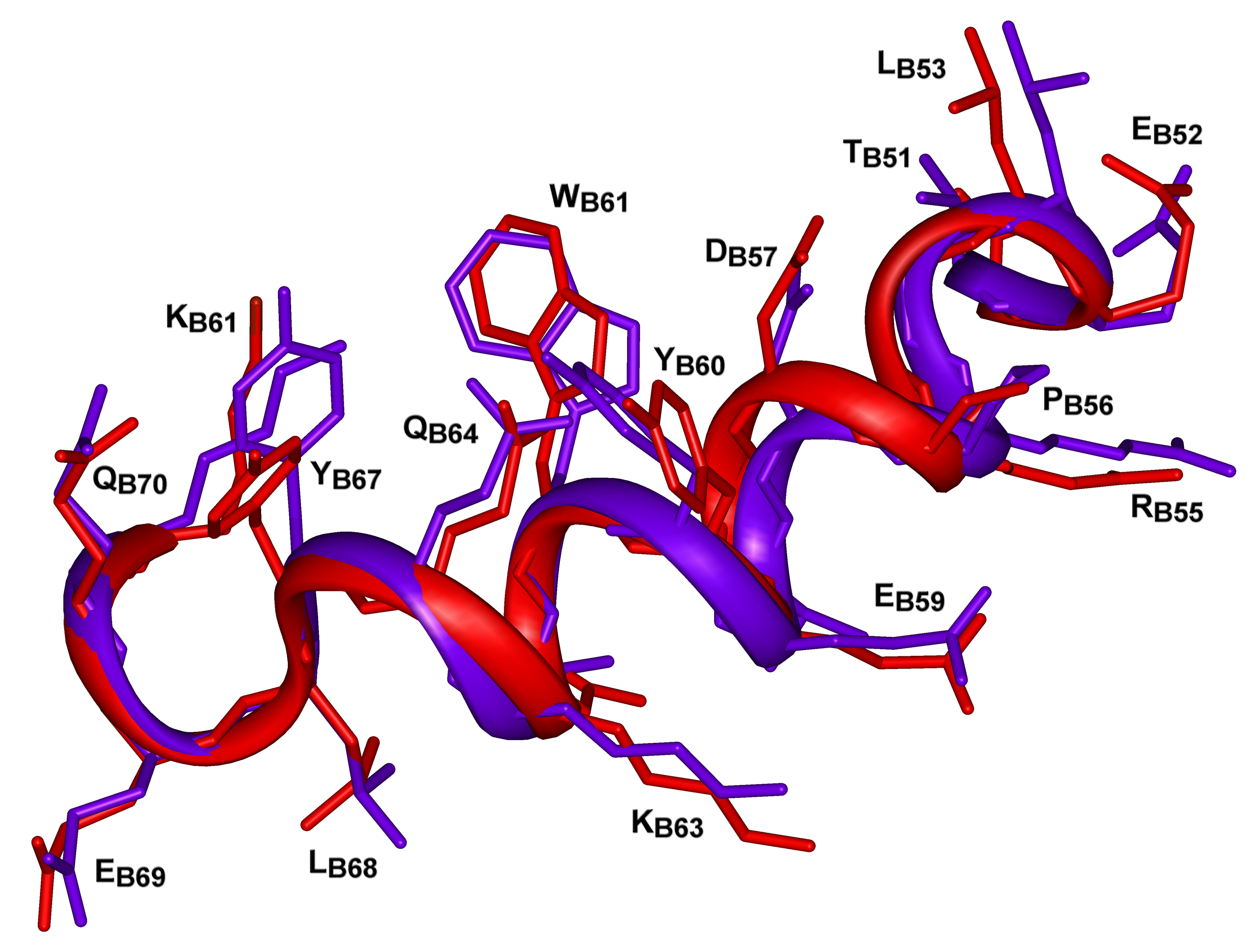

Supplement: S3 Fig — Superposition of two molecular models of the H2-Aj protein obtained by the homology modelling approach from atomic coordinates of 1MUJ (red) and 2P24 (purple). Fragments B51-B71 of the β-chain are shown. The sequence of H2-Aj differs from the 1MUJ (but not from 2P24) by deletion of residues 65 and 66. (TIF) [file pgen.1005672.s003.tif]

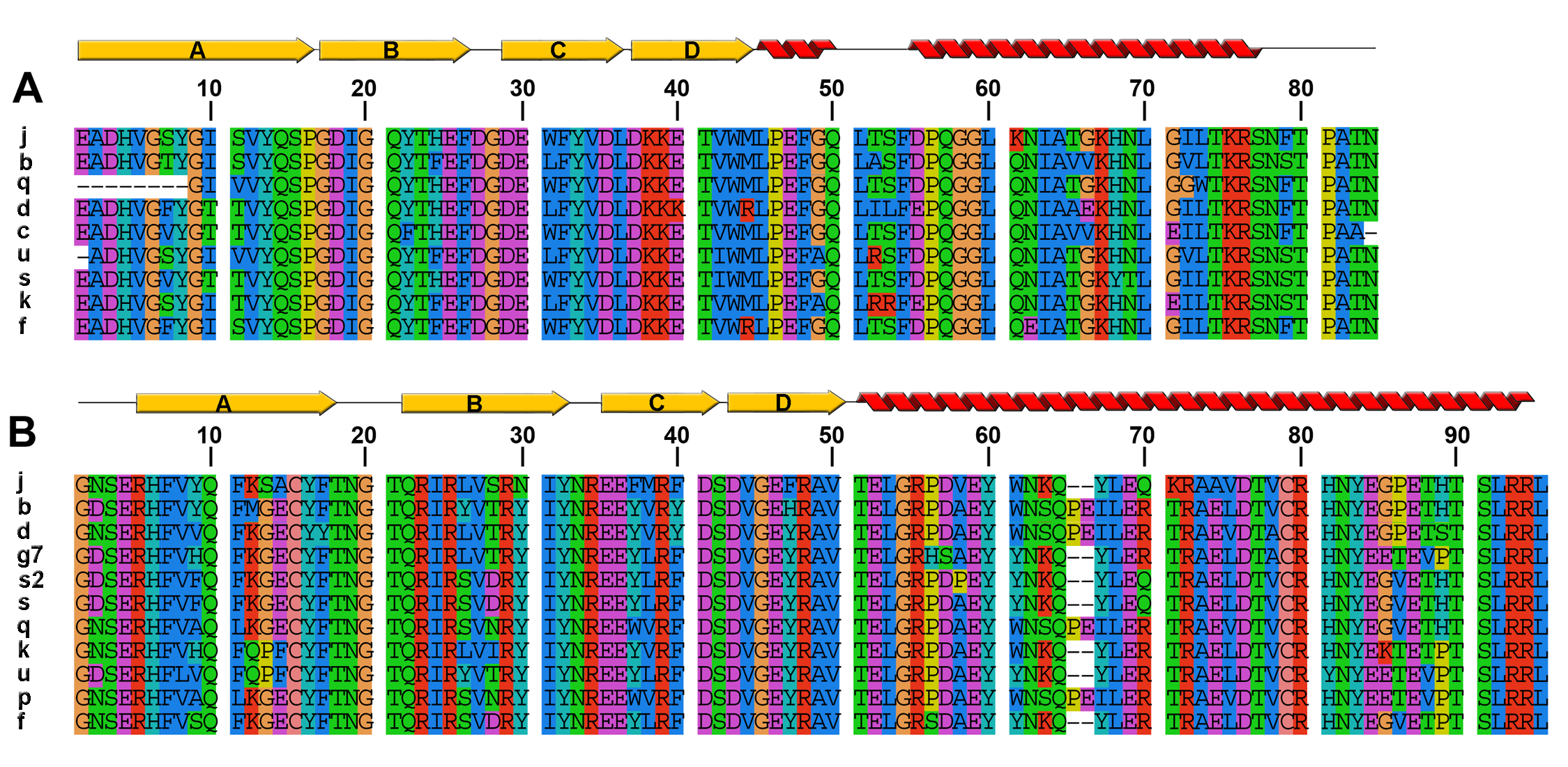

Supplement: S4 Fig — Alignment of α1 and β1 polymorphic domains of the H2-Aj α- (A) and β- (B) chain with annotated haplotypes of inbred mouse strains (left column). Sequences were taken from IMGT (http://www.imtg.org) database. The features of secondary structure are given according to (Lefranc et al., Develop. Compar. Immun, 2005; 29: 917–938). Yellow arrows mark four beta strands, the red spiral is α-helix. Multiple sequence alignment was made using ClustalW2 program (Larkin et al., Bioinformatics 2007, 23: 2947–2948). (TIF) [file pgen.1005672.s004.tif]

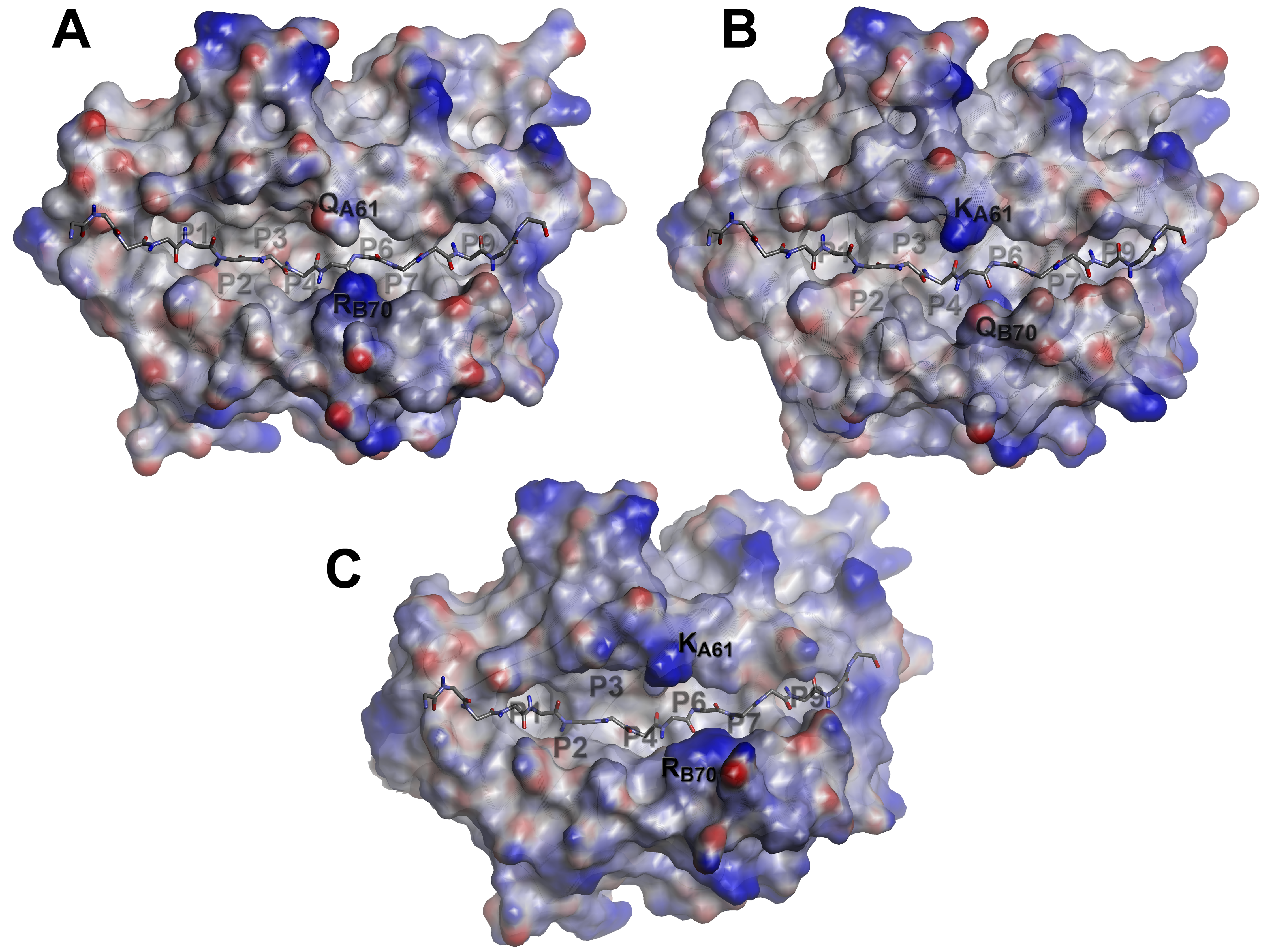

Supplement: S5 Fig — Solvent accessible surface representation of the MHC-binding groove in H2-Ab (A), H2-Aj (B) and hybrid H2-Ajb* (C) molecules. Molecular surfaces are colored according interpolated electrostatic potential. Heavy atoms of the CLIP peptide are shown as sticks colored grey (carbon), blue (nitrogen) and red (oxygen). AA residues in positions α61 and β70 may form a salt bridge and are potentially available for interaction with TCR. (TIF) [file pgen.1005672.s005.tif]

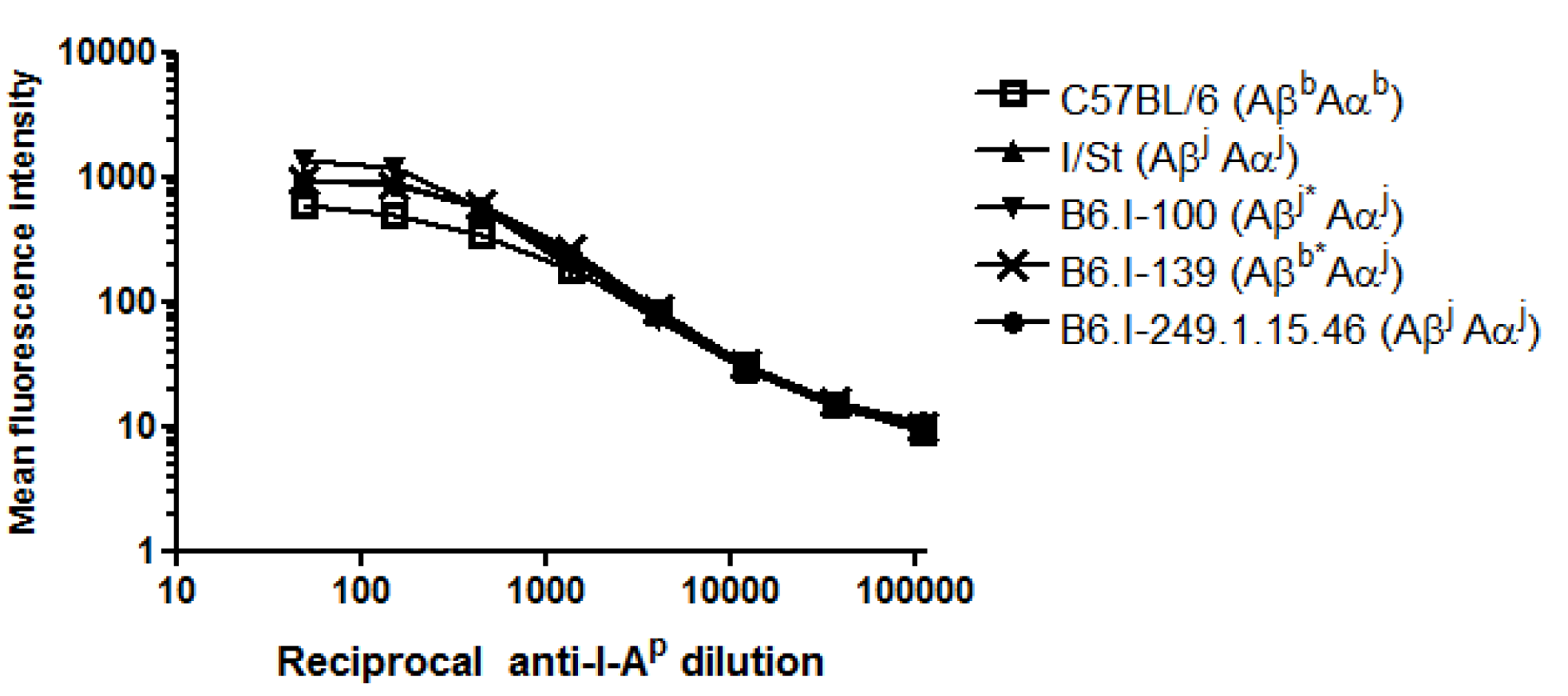

Supplement: S6 Fig — Spleen cells were stained with serial dilutions of the anti-I-Ap mAb (clone 7–16.7; BD Biosciences) and analyzed by flow cytometry. (TIF) [file pgen.1005672.s006.tif]
