# Supplementary material for: The QTL within the H2 Complex Involved in the Control of Tuberculosis Infection in Mice Is the Classical Class II H2-Ab1 Gene
Source: PLoS Genet. 2015 Nov 30;11(11):e1005672. doi: 10.1371/journal.pgen.1005672 (PMC4664271; doi:10.1371/journal.pgen.1005672)
Supplement: S1 Table — (DOC) [file pgen.1005672.s007.doc]

**S1 Table**
