# Supplementary material for: The QTL within the H2 Complex Involved in the Control of Tuberculosis Infection in Mice Is the Classical Class II H2-Ab1 Gene
Source: PLoS Genet. 2015 Nov 30;11(11):e1005672. doi: 10.1371/journal.pgen.1005672 (PMC4664271; doi:10.1371/journal.pgen.1005672)
Supplement: S2 Table — (DOCX) [file pgen.1005672.s008.docx]

**S2 Table**

| **Mouse strain** | **CD4^+^ total (millions per lung)** | **CD4^+^ IFN-γ^+^ (millions per lung)** | **CD4^+^ IFN-γ^+^ MFI)** |
| --- | --- | --- | --- |
| C57BL/6 | 4.4 ± 1.2 | 0.24 ± 0.08* | 304 ±10* |
| I/St | 3.9 ± 0.3 | 0.05 ± 0.00 | 135 ± 44 |
| B6.I-100 | 4.5 ± 0.7 | 0.08 ± 0.01 | 153 ± 12 |
| B6.I-139 | 3.2 ± 0.6 | 0.13 ± 0.03* | 252 ± 45* |
| B6.I-249.1.15.46 | 2.9 ± 0.7 | 0.04 ± 0.01 | 127 ± 35 |

^#^ Summary of data obtained by FACS analyses, see legend to Fig. 5C for details. MFI- mean fluorescence intensity. **P* < 0.05 compared to I/St, B6.I-100 and B6.I-249.1.15.46 mice, ANOVA. The total numbers of CD4^+^ T-cells did not differ between mouse strains.
